# Bioprosthetic Aortic Valve Degeneration After TAVR and SAVR: Incidence, Diagnosis, Predictors, and Management

**DOI:** 10.3390/jcdd11120384

**Published:** 2024-11-30

**Authors:** Nadera N. Bismee, Niloofar Javadi, Ahmed Khedr, Fatma Omar, Kamal Awad, Mohammed Tiseer Abbas, Isabel G. Scalia, Milagros Pereyra, George Bcharah, Juan M. Farina, Chadi Ayoub, Kristen A. Sell-Dottin, Reza Arsanjani

**Affiliations:** 1 Department of Cardiovascular Medicine, Mayo Clinic, Phoenix, AZ 85054, USA; 2 Department of Cardiovascular Surgery, Mayo Clinic, Phoenix, AZ 85054, USA; 3 Mayo Clinic Alix School of Medicine, Phoenix, AZ 85054, USA

**Keywords:** bioprosthetic aortic valve, structural valve degeneration, aortic stenosis, aortic valve replacement

## Abstract

Bioprosthetic aortic valve degeneration (BAVD) is a significant clinical concern following both transcatheter aortic valve replacement (TAVR) and surgical aortic valve replacement (SAVR). The increasing use of bioprosthetic valves in aortic valve replacement in younger patients and the subsequent rise in cases of BAVD are acknowledged in this review which aims to provide a comprehensive overview of the incidence, diagnosis, predictors, and management of BAVD. Based on a thorough review of the existing literature, this article provides an updated overview of the biological mechanisms underlying valve degeneration, including calcification, structural deterioration, and inflammatory processes and addresses the various risk factors contributing to BAVD, such as patient demographics, comorbidities, and procedural variables. The difficulties in early detection and accurate diagnosis of BAVD are discussed with an emphasis on the need for improved imaging techniques. The incidence and progression of BAVD in patients undergoing TAVR versus SAVR are compared, providing insights into the differences and similarities between the two procedures and procedural impacts on valve longevity. The current strategies for managing BAVD, including re-intervention options of redo surgery and valve-in-valve TAVR, along with emerging treatments are discussed. The controversies in the existing literature are highlighted to offer directions for future investigations to enhance the understanding and management of BAVD.

## 1. Introduction

Aortic valve replacement (AVR) through surgery or catheter-based interventions is the first-line therapy for patients with severe aortic valve disease [1,2]. There are two main types of heart valve prostheses that can be utilized: mechanical prosthetic valves and biological tissue valves [3]. Unlike mechanical valves, biological tissue valves do not require long-term anticoagulation. However, they do not last as long as their mechanical counterparts and are more susceptible to degeneration and eventual dysfunction [3,4]. Tissue bioprostheses of animal origin are more widely used, which are usually made from bovine pericardium or porcine valve leaflets. Homografts are less frequently used and are composed of human tissue from the aortic root or pulmonary trunk [1,3].

The increase in aortic valve replacement procedures and the increase in the use of bioprosthetic valves accounts for the increase in the incidence of valve degeneration and highlights the importance of ongoing surveillance and research to optimize patient outcomes. While transthoracic echocardiogram remains the primary modality of determining valve function, results from new imaging modalities seem promising in diagnosing early valve dysfunction [5]. The degenerative process, influenced by various risk factors such as patient age, valve type, and underlying comorbidities, underscores the complexity of managing these prosthetic devices [6,7,8].

In the management of bioprosthetic aortic valve degeneration, redo surgical aortic valve replacement has traditionally been considered the gold standard method, but it is increasingly being substituted by transcatheter aortic valve implantation (TAVR) in eligible patient population [9]. Multiple factors need to be considered to decide between SAVR and TAVR for reinterventions in cases of degeneration [10]. In this review article, we highlight the underlying pathophysiology, the factors contributing to bioprosthetic valve degeneration, and potential therapeutic options in cases of degeneration.

## 2. Prevalence of Bioprosthetic Aortic Valve Replacement

Globally, there are 200,000 to 400,000 heart valve replacement surgeries performed annually. Aortic valve replacement accounts for the majority of surgical interventions, followed by mitral and tricuspid valve replacements [3,11]. Over the last 15 years, bioprosthetic aortic valve (BAV) implantation has increasingly become the treatment of choice for patients in need of AVR relative to mechanical prostheses [6]. Each year in the United States, around 90,000 surgical AVR are conducted, with more than three-quarters of these involving BAV. The rapid increase in the implantation of BAV can be attributed to patient preference, the rising prevalence of valve disease in an aging population, and the emergence of TAVR.

Over 276,000 TAVR procedures have been performed in less than a decade since receiving U.S. Food and Drug Administration approval in 2011 [4,12]. It is anticipated that by 2050, the number of prosthetic valve implantations annually will triple, attributed to the aging population and the growing prevalence of transcatheter procedures, with estimates reaching 850,000 per year [2]. TAVR has emerged as a valid option for patients with severe aortic stenosis who are at intermediate to high/prohibitive surgical risk [5,6,7,8,9]. Moreover, multiple trials evaluated TAVR for treating patients who are at low surgical risk with positive results [6,11,12]. Mack, M. J. et al. (2023) conducted a randomized controlled trial involving 1000 patients and found that there is no significant difference in the 5-year primary end points between low-risk patients with severe, symptomatic stenosis who underwent TAVR or SAVR [13]. All transcatheter valves are BAV, and the arrival of TAVR, coupled with the growing field of valve-in-valve procedures in the case of structural valve degeneration (SVD) [14], has further stimulated the increased use of aortic bioprostheses in patients with aortic valve diseases [3].

The decision on whether to opt for a mechanical valve or BAV for patients undergoing valve replacement is complex, considering various prosthesis types and patient factors. Surgical BAV, made of leaflets from bovine pericardium, porcine valve leaflets, and occasionally from human tissue, are increasingly favored for their favorable hemodynamic profile, acceptable durability (mainly in recent generations of bioprosthetic valves), and low thrombogenicity [15]. However, BAV utility is limited by SVD, a phenomenon characterized by deterioration of the prosthetic valve leaflets or supporting structures with eventual associated valve hemodynamic dysfunction, manifested as either stenosis or regurgitation [14].

The incidence of SVD in BAV is rare in patients who have undergone SAVR within 5 years, but it increases substantially beyond 7 to 8 years after implantation [15]. The rate of SVD requiring valve re-intervention reaches 20% at 10 years and 30% at 15 years [16,17,18,19]. The durability of the newer generations of BAV has improved, with incidence of SVD of 2% to 10% at 10 years, 10% to 20% at 15 years, and 40% at 20 years after surgery [20,21,22,23]. However, it should be noted that the incidence of re-operation for SVD may significantly underestimate the true incidence of SVD. Indeed, several studies have reported that about 25–35% of patients with a BAV in the aortic position present with some degree of valve degeneration/dysfunction at the Doppler echocardiographic exam within 10 years [21,24,25,26]. Some studies have also identified higher rates of SVD, including hemodynamic changes, in up to 10% of patients in 5 years and 30% of patients in 10 years after surgery. The variability in the studies indicate that these do not represent the true rates of SVD which can be assumed to be higher than reported [13,14,27].

With the above-mentioned increase in the use of BAV, there is a growing need for a better understanding of the SVD phenomena, its risk factors, and effective management strategies.

## 3. Pathophysiology of Degeneration

**Figure 1:**
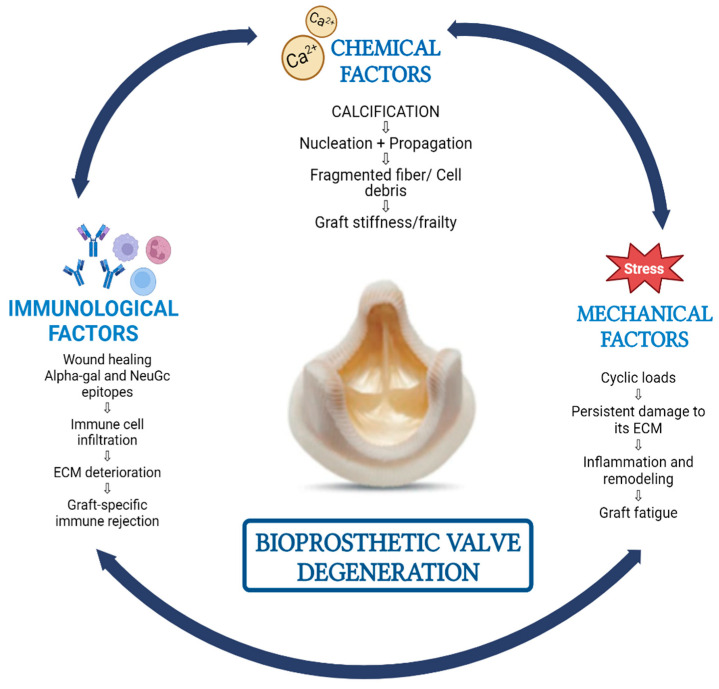
Summary of the potential pathophysiological mechanisms of bioprosthetic aortic valve degeneration.

Although the major determinant of BAV durability is SVD, the exact pathophysiology is yet to be understood [11]. SVD is defined as a multifactorial process which can be influenced broadly by chemical, mechanical, and immunological factors [27] (Figure 1). Calcification is a common outcome of all these processes and the primary cause of BAV injury and dysfunction. It can lead to increasing thickness, stiffness, and obstruction of the cusps, as well as fragility and tears in the leaflets [28,29,30]. Bovine pericardial valves commonly exhibit stenosis as a mode of SVD, while porcine valves are more prone to develop tears in the leaflets, often leading to regurgitation [6]. On the other hand, a small number of BAVs seem to deteriorate without any apparent histological evidence of calcification. In these instances, the notable histological characteristics were significant thickening of the leaflets, fluid accumulation, and disruption of the normal collagen structure [5].

The SVD of BAV is a complex process involving both passive and active inter-related mechanisms [27]. Passive deterioration is hypothesized to be due to the absence of living cells in the graft, which are typically responsible for maintaining valve homeostasis and repairing damaged extracellular matrix (ECM). Graft fatigue is the primary driving force behind this passive deterioration, resulting from persistent damage to its ECM under the influence of repeated cyclic loads [31]. Dystrophic calcification is also a passive mechanism, resulting from calcium phosphate (CaP) precipitation on the graft surface, caused by an abundance of potential nucleation foci, like fragmented fibers or cell debris [3]. Mahjoub, H. et al. (2015), in an epidemiological study, demonstrated that higher CaP product in the serum directly correlates with BAV calcification [25].

Active mechanisms of BAV deterioration are mediated mainly by recipient cells. Multiple studies have demonstrated that BAVs can provoke humoral and cellular immune responses [3,32,33,34,35]. BAVs are subjected to infiltration by immune cells producing proteolytic enzymes, calcium-binding proteins, and reactive oxygen species [35,36,37]. Under certain conditions, the inflammatory reaction within the BAV results in an atherosclerosis-like phenotype, ultimately leading to graft dysfunction [38]. BAV tissues can also induce the production of human antibodies against α-Gal (a carbohydrate molecule found on the surface of cells in many mammals but not in humans) and NeuGc (a sialic acid derivative found in most mammals but not in humans due to a mutation in the CMAH gene) [32,39]. Antibodies against both epitopes can enhance immune cell recruitment and associated calcification. Both active and passive mechanisms of SVD can occur simultaneously, reinforcing each other. For instance, mechanical stress induces the proteolytic cleavage and delamination of graft ECM, facilitating immune cell infiltration and ECM deterioration. This consequently leads to areas susceptible to mechanical destruction. [3].

About 15% of patients who have undergone biological AVR can develop valve thrombosis during the initial postprocedural period [40,41,42]. This may instigate inflammation and lead to subsequent fibrocalcific remodeling of the valve leaflets even if it is subclinical and has been successfully managed with anticoagulant agents [33]. Additional research will help ascertain whether valve thrombosis in the early post-AVR phase contributes to an accelerated occurrence of SVD [6].

## 4. Risk Factors for Bioprosthetic Valve Degeneration

Multiple risk factors for bioprosthetic SVD have been identified over the years. The main factors associated with SVD following AVR can be divided into three groups: demographics (age and sex), cardiovascular risk or comorbid conditions (dyslipidemia, smoking, hypertension, diabetes mellitus, metabolic syndrome, raised body mass index and body surface area, and renal disease), and factors related to the valve itself (prosthesis brand and size and patient–prosthesis mismatch) [6,7,8].

Ochi, A. et al. (2020) conducted a meta-analysis of twenty-nine observational studies including 25,490 patients, of whom 981 developed SVD over a mean follow-up time of 18.5 years. Through quantitative analysis, four factors including age, patient–prosthesis mismatch, body surface area, and smoking were found to be significant determinants of bioprosthetic SVD. The authors concluded that as age increases by 1 year, the risk of SVD decreases by 9% (HR 0.91; 95% CI 0.89−0.94; *p* < 0.0001), and also stated that patient–prosthesis mismatch (PPM) (HR 1.95; 95% CI 1.56–2.43; *p* < 0.001), a raised BSA (HR 1.77; 95% CI 1.04–3.01; *p* = 0.0339), and smoking (HR 2.28; 95% CI 1.37–3.79; *p* = 0.0015) were associated with a significantly increased risk for degeneration [43]. These risk factors share the commonality of increasing the flow rate across the valve prosthesis, inducing mechanical stress. Younger patients, those with a higher body surface area, and individuals who smoke all experience an elevated metabolic rate, leading to a higher cardiac output [8]. The increased flows caused by the elevated cardiac output create shearing forces on valve leaflets, which may play a significant role in the development of bioprosthetic SVD [43].

In cases of PPM in need of a reintervention, intentional ring fracturing of BAVs (cracking technique) could be an effective method to allow for further expansion of the new implanted valve during a ViV TAVR. This could lead to reduced transvalvular gradients and increased EOA postoperatively. For certain patients, this technique might help address paravalvular leaks (PVLs) in conjunction with deteriorated BAVs. Two key factors need to be considered: the specific model of the BAV (which determines the feasibility of ring fracturing) and the morphology and extent of the leak (which will influence the success of sealing when using ViV TAVR followed by ring fracturing) [44]. Further studies are needed to assess the effectiveness of this approach and identify appropriate conditions for its use. 

According to previous studies of BAV outcomes, age at the time of valve implantation has been the most important patient-related factor in determining the bioprosthesis valve longevity [6,45]. It is well known that younger patients are more susceptible to SVD, which is one of the reasons why prosthesis choice recommendations are often mainly based on age [1,2]. Through continuous and categorical analysis, the meta-analysis reiterated that advancing age is a protective factor [43]. At 10-year follow-up after SAVR, the rate of SVD is usually <10% in elderly patients, whereas it rises to 20% to 30% in patients < 40 years of age [46,47].

On the other hand, Nitsche, C. et al. (2020), in a recent observational study that included 466 patients undergoing SAVR and followed them for a median of 112.3 months, contradicted this concept [11]. The study demonstrated that patients younger than 70 years were not affected by faster SVD of BAVs; in fact, there was a strong trend toward shorter time to SVD in patients older than 80 years. The only risk factors identified in the study were the use of porcine tissue valves, renal impairment, hypertension, and PPM [11]. Interestingly, most studies that used a definition of SVD based on imaging criteria rather than reintervention found no association between age and SVD [28]. 

The risk associated with the gender is controversial with some studies showing male sex to be associated with accelerated SVD [8,48]; whereas, according to other studies, to the contrary, female sex is a risk factor for SVD [47,49].

The AHA/ACC 2014 *Guideline on Perioperative Cardiovascular Evaluation and Management of Patients Undergoing Noncardiac Surgery* and ESC/EACTS 2012 Guidelines on the management of valvular heart disease acknowledge the increased risk of accelerated SVD in those with renal disease [50,51]. This is expected due to the changes in calcium metabolism, anemia, and arteriovenous fistula, which can increase cardiac output [43]. Renal impairment often leads to dysregulation of phospho-calcic metabolism, resulting in a rapid progression of valvular degeneration. Patients with renal impairment have a high co-prevalence of arterial hypertension, which could further accelerate SVD by increasing diastolic closure stress on the prosthesis [6,11]. 

The presence of histological evidence of atherosclerotic-like processes in bioprostheses with SVD, and the similarity of SVD to native aortic stenosis, suggests that atherosclerotic risk factors could also be risk factors for bioprosthetic SVD, and recent studies have shown a strong association between atherosclerotic risk factors and the development of SVD [6,15,43,52]. These risk factors include metabolic syndrome, diabetes, an increased proportion of small, dense LDL particles, lipoprotein-associated phospholipase A2 (Lp-PLA2), apoB/apoA ratio, lipoprotein(a), and proprotein convertase subtilisin/kexin 9 (PCSK9) [28,53]. Such findings support the role of lipid-mediated inflammation in the calcific degeneration of bioprosthetic valve leaflets [15].

Dysfunctional BAVs exhibit significant lipid deposition and contain foam cells, which are specific to atherosclerosis. Prior studies demonstrated the association between SVD and impaired lipid metabolism. A high risk of SVD has been associated with an increased ratio of LDL/high-density lipoprotein, reflecting the balance between proatherogenic and antiatherogenic lipoproteins. A retrospective multicenter study involving 210 cases demonstrated an association between high serum Lp(a) and BAV degeneration. Median serum levels of Lp(a) were significantly higher in patients affected by degeneration versus non-affected cases: 50.0 (IQR 72.0) vs. 15.6 (IQR 48.6) mg/dL, *p* = 0.002, in patients who underwent a BAV replacement between 1 January 2010 and 31 December 2020 [53].

Moreover, SVD has been correlated with insulin resistance, increased lipoprotein-associated phospholipase A2 activity, and higher levels of subtilisin-kexin type 9 proprotein convertase. The mechanism behind LDL deposition and oxidation in BAVs is not fully understood. It is possible that LDL may interact with glycosaminoglycans, resembling native aortic stenosis and atherosclerotic lesions. Oxidation of LDL is likely mediated by infiltrating inflammatory cells, which contributes to the release of reactive oxygen species into the BAV environment [3]. 

Lorusso et al. in his study involving twelve Italian centers which included 6184 patients undergoing bioprosthetic implants from 1988 to 2009, concluded type 2 DM to be a strong predictor of SVD (hazard ratio 2.39 [95% confidence interval 2.28–3.52]). It appeared that the risk is greater for patients treated with insulin, for those with Hb A1c > 6.5%, and for those with FPG > 142 mg/dL. Therefore, even patients with apparently well-controlled or mild DM fall under the high-risk group for SVD [24].

## 5. Challenges in Screening and Diagnosis

The durability of BAV is mainly determined by SVD, which was previously defined in many studies as the need for reoperation largely due to a lack of regular echocardiographic follow-up [16]. This approach may exclude patients that are ineligible for reoperation, resulting in an underestimation of the incidence of SVD. More recently, SVD has been defined using echocardiographic criteria, based on changes in trans-prosthetic gradients and the severity of regurgitation [11,21,54]. 

Comparing the durability of bioprostheses is challenging due to inconsistent definitions of SVD [30]. The need for reoperation is often used to define SVD but is unreliable because reinterventions might occur for reasons other than SVD, or SVD might go undetected without echocardiographic follow-up. High-risk patients might not undergo repeat surgery, underestimating SVD rates [55]. Since 2008, surgical guidelines have recommended defining SVD through clinically detectable measures beyond reoperation needs, such as echocardiographic criteria. Zoghbi et al. (2009) provided recommendations for evaluating prosthetic valves using echocardiography and Doppler ultrasound, defining possible and significant stenosis based on specific velocity, gradient, and orifice area metrics. They defined possible stenosis as a peak prosthetic aortic jet velocity of 3–4 m/s, a mean gradient of 20–35 mmHg, and an effective orifice area of 0.8–1.2 cm^2^. Significant stenosis was defined as a peak prosthetic aortic jet velocity > 4 m/s, a mean gradient > 35 mmHg, and an effective orifice area < 0.8 cm^2^ [29]. 

The 2009 American Society of Echocardiography and VARC-2 recommendations provide specific criteria for evaluating BAVs, but definitions still vary. The European Association for Cardiovascular Imaging and the European Association of Percutaneous Cardiovascular Interventions suggest incorporating hemodynamic and morphological criteria for a more accurate assessment [30]. 

The VARC-2 criteria define SVD as valve-related dysfunction (mean aortic gradient ≥ 20 mmHg, effective orifice area ≤ 0.9–1.1 cm^2^, dimensionless valve index < 0.35, or moderate/severe prosthetic valve regurgitation) or the need for a repeat procedure (TAVR or SAVR) [29]. Lancellotti et al. suggested incorporating stress echocardiography to detect gradient increases (10–19 mmHg for possible obstruction; ≥20 mmHg for significant obstruction) [3]. Bourguignon et al. recently defined SVD using strict echocardiographic criteria for severe aortic stenosis (mean transvalvular gradient > 40 mmHg) and severe aortic regurgitation (effective regurgitant orifice area > 0.30 cm^2^; vena contracta > 0.6 cm) [30]. 

Transthoracic echocardiography (TTE) is the recommended primary imaging technique in the initial evaluation of patients with known or suspected SVD and also during follow-up for monitoring prosthetic valve function according to the AHA/ACC Guideline for the Management of Patients with Valvular Heart Disease [56]. The European Association of Cardiovascular Imaging recommends baseline TTE evaluation within 30 days of implantation, at 1 year, and then annually afterwards [1,57]. Earlier follow-up studies should be considered when changes in clinical symptoms or new signs suggesting valve dysfunction occur. An initial TTE examination performed 6 weeks to 3 months after prosthetic valve implantation is recommended to assess the results of surgery and serve as a baseline for comparison should deterioration occur later [6,15,51].

**Figure 2:**
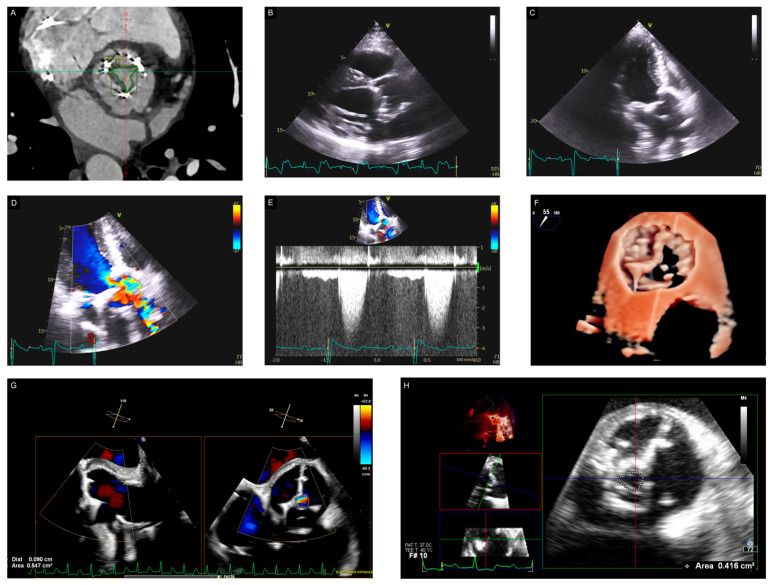
A 38-year-old male with a history of a bicuspid aortic valve underwent SAVR at the age of 30. A CT scan (Panel **A**) demonstrated thickening of the aortic valve prosthesis leaflets, most notably the noncoronary-facing and right coronary-facing leaflets, both of which are hypomobile. Significant thickening extends to the base of the right and noncoronary cusps at the valve plane. A TTE examination showed that the aortic valve prosthesis leaflets were calcified with reduced opening from parasternal and apical views (Panels **B**,**C**). The systolic mean Doppler gradient was 28 mmHg (EOA 1.4 cm^2^) (Panels **D**,**E**). A TEE confirmed the severity of the stenosis (Panels **F**–**H**); all the leaflets had markedly reduced opening. They were calcified except for the leaflet towards the left coronary cusp, which was not well visualized. By planimetry, the aortic valve area was between 0.4 and 0.5 cm^2^, indicative of severe aortic stenosis.

TTE allows accurate assessment of valve structure and function, identifies concurrent valve disease, and highlights related abnormalities like aortic dilation. A complete echocardiography evaluation involves two-dimensional imaging of the prosthetic valve, evaluation of valve leaflet morphology and mobility, measurement of the trans-prosthetic gradients and effective orifice area (EOA), and precise estimation of the location and degree of regurgitation [15] (Figure 2). Doppler echocardiography provides accurate noninvasive determination of valve hemodynamics. For stenotic disease, critical measurements include maximum velocity, mean gradient, and valve area. For regurgitant lesions, calculation of regurgitant orifice area, volume, and fraction is performed. If possible, this is conducted in the context of a multiparameter severity grade based on color Doppler imaging, continuous- and pulsed-wave Doppler recordings, and the presence or absence of distal flow reversals [58]. Overestimation or underestimation of the severity of valve regurgitation may result if the image or Doppler data quality is suboptimal.

Nonetheless, first-line screening of prosthetic valves with TTE has remarkable limitations, as gradients could be normal despite valve thrombosis which may cause valve dysfunction and degeneration. Acoustic shadowing from the TAVR device may hinder adequate visualization of the TAVR device leaflets and transvalvular gradients, depending on the type and size of the implanted valve. Therefore, unless there is a significant change from baseline gradients, TTE may lack sufficient sensitivity in detecting early hemodynamic changes. 

Transesophageal echocardiography (TEE) is reported to have comparable sensitivity to CT in the detection of leaflet thickening, thrombotic appositions, or restricted leaflet mobility. Although it is more invasive than TTE or CT, it may be appropriate when TTE images are suboptimal and in patients at a higher risk of contrast-induced nephropathy. To avoid the acoustic shadowing created by the bioprosthetic scaffold, a deeper TOE longitudinal view with slight anterior flexion of the probe is often recommended [58,59]. 

Multidetector computed tomography (MDCT) may also be valuable in evaluating the valve leaflet thickening/calcification and reduced mobility [15]. Cardiac CT, due to its high spatial resolution and high sensitivity for calcification, can provide an accurate structural assessment of valve integrity and calcification. It can accurately locate and differentiate thrombus from pannus by measuring Hounsfield units—a scale for radiodensity on CT scans. Additionally, it can identify HALT (hypoattenuation leading to leaflet thickening), RLM (reduced leaflet motion), and HAM (hypoattenuation affecting motion). In cases of HALT and HAM, anticoagulation can help dissolve thrombus formation on the valve leaflets, reducing the chance of accelerated SVD and thus prolonging the functional life of the valve. Cardiac CT is now frequently used to assess prosthetic aortic valve dysfunction, complementing the hemodynamic information provided by echocardiography [60].

Magnetic Resonance Imaging (MRI) may be a useful alternative to echocardiography in patients with SVD for assessing the hemodynamics of the BAV, especially when echocardiography is limited by imaging windows. Although BAVs can cause artifacts on MR images, it has been shown to be relatively safe for imaging at both 1.5-T and 3.0-T for several valve types. However, in both normal and dysfunctional BAVs, the effective orifice area calculated from MRI has been shown to correlate to the effective orifice area calculated using echocardiography [61,62,63,64,65].

Although noninvasive testing is now able to provide the required anatomic and hemodynamic information in most patients with SVD, there remains a subset of patients for whom hemodynamic catheterization is essential to ensure the correct treatment decision is made. Hemodynamic cardiac catheterization, which includes direct intracardiac measurements of transvalvular pressure gradients and cardiac output, can provide crucial clinical information, especially when noninvasive tests yield inconclusive results or when they conflict with clinical observations [60,66]. Contrast angiography can be helpful for a semiquantitative assessment of the severity of regurgitation, especially when noninvasive results conflict with the physical examination [58].

In patients in which more sophisticated tests may be required, both the performance and interpretation of these diagnostic tests require meticulous attention to detail and expertise in the field. Because echocardiography remains the mainstay of the initial evaluation of all patients with SVD, it is recommended that the laboratory be an Intersocietal Accreditation Commission (IAC)-accredited program [58].

^18^F-fluoride PET-CT has been identified as the first noninvasive technique capable of detecting early SVD and predicting future valve dysfunction. According to a multimodality prospective imaging study, increased radioisotope uptake was consistently demonstrated in all the failed BAVs, with PET activity colocalizing to areas of calcification, pannus, thrombus, and disrupted tissue architecture on histology. ^18^F-fluoride PET identified early SVD beyond the resolution of conventional assessments and predicted the development of new valvular dysfunction within the 2-year follow-up period. It was revealed that patients with increased radioisotope uptake experienced more rapid deterioration in valve function compared with those without (annualized change in peak transvalvular velocity 0.30 [IQR: 0.13 to 0.61] vs. 0.01 [IQR: −0.05 to 0.16] ms^−1^/year; *p* < 0.001). ^18^F-fluoride uptake was an independent predictor of deteriorating bioprosthetic valve performance, surpassing all other variables, including valve type and age, echocardiographic, and CT findings. The study further validates the use of baseline ^18^F-fluoride PET in identifying patients at risk of developing SVD. This novel imaging approach could have the potential to transform our understanding of SVD and the way we monitor and manage patients living with BAVs [5]. 

## 6. Bioprosthetic Valve Degeneration Following TAVR and SAVR

Among patients with aortic stenosis, 1 million are eligible for TAVR and 1.9 million patients are eligible for SAVR [10]. Surgery has always been the gold standard management for AS. However, TAVR was first introduced in 1965, and was first performed on a patient in 2002 [67]. Since then, the awareness and prevalence of this procedure which was first approved for commercial use in 2007 in Europe and 2011 in the United States has significantly increased [9]. Lately, numerous studies have emerged comparing TAVR to SAVR and indications for each procedure continue to rise. TAVR procedures have surpassed SAVR procedures in number over recent years. The potential use of TAVR in younger and lower risk patients has raised concerns regarding the transcatheter heart valve longevity and its effects on future reinterventions [68].

As TAVR expands to younger, lower-risk patients, various anatomical and clinical challenges will be encountered. While transfemoral access is the gold standard and works for most aortic stenosis patients, some candidates may not be ideal due to small vessel size, severe peripheral artery disease, or extensive vessel and aortic tortuosity. Complex aortic anatomy, such as a horizontal aorta or an aortic aneurysm, can also hinder device delivery and accurate implantation. For patients with bicuspid aortic valve, SAVR remains the preferred treatment over TAVR due to higher rates of complications like PVL, pacemaker implantation, valve malposition, annular rupture, and mortality. Additionally, coronary anatomy poses challenges, particularly when the distance between the aortic annulus and coronary ostia is short, increasing the risk of coronary obstruction, thus favoring SAVR over TAVR in such cases [69].

The PARTNER 2 trial published in 2020 compared outcomes between SAVR and two different generations of the TAVR devices, the SAPIEN XT TAVR (2nd generation) and the SAPIEN 3 TAVR (3rd generation). Patients from two different previous trials (the PARTNER 2A randomized trial and the PARTNER 2 SAPIEN 3 observational study, for a total of 2329 patients) were enrolled in this study. To be included in the study, patients had to have symptomatic, severe AS and be classified as having an intermediate risk for 30-day surgical mortality. When comparing SAVR with the SAPEN XT TAVR, it was found that the 5-year Kaplan–Meier cumulative rates of SVD were significantly higher in the SAPIEN XT TAVR group vs. the SAVR group (9.5%; 95% CI: 7.0% to 12.7% vs. 3.5%; 95% CI: 2.1% to 5.8%, respectively, *p* < 0.001).

In comparing the SAVR with the SAPIEN 3 TAVR group, the 5-year Kaplan–Meier cumulative rates of SVD were similar (3.9%; 95% CI: 2.5% to 6.0% vs. 3.5%; 95% CI: 2.1% to 5.8%, respectively; *p* = 0.65). This study also revealed a significant association between the female sex (*p* < 0.01) and smaller transcatheter valve size (*p* < 0.01) and development of SVD among the SAPIEN XT TAVR cohort. It also revealed that younger age (*p* = 0.02), the female sex (*p* < 0.01), and diabetes (*p* = 0.01) served as significant predicators for SVD in the SAPIEN 3 TAVR cohort. No factors were found to be significantly associated with SVD in the SAVR cohort [70]. Conversely, another multicenter, randomized trial comparing the use of the SAPIEN 3 TAVR with SAVR in 1000 patients with severe, symptomatic AS at low surgical risk found that the incidence of bioprosthetic valve failure (BVF) related to SVD was 1.4% in the TAVR group vs. 2.0% in the surgery group (BVF was evaluated by a team of 3 senior echocardiographers, according to the Valve Academic Research Consortium (VARC) 3 criteria) [13].

Other studies have found no significant difference in the risk of SVD between SAVR and TAVR. In a prospective observational cohort study published in October 2021, patients with AS who had undergone TAVR (1 month, 2 years, or 5 years ago) using a balloon-expandable or self-expanding bio prosthesis were compared with patients that had undergone SAVR. This study was conducted at three high-volume TAVR centers between September 2016 and November 2019. All participants did not have established clinical evidence of bioprosthetic valve degeneration at the start. Each patient underwent clinical assessment, echocardiography, hybrid ^18^F-NaF PET [71,72], and CT angiography at baseline with annual echocardiography after. SVD occurred in 47 patients with TAVR and 51 patients with SAVR with the median age being higher in TAVR patients (82 (76–86) vs. 72 (70–77) years, respectively; *p* < 0.001). The time from valve replacement to imaging was similar (24 (24–60) vs. 24 (24–60) months; *p* = 0.91). Results showed patients with TAVR to have a lower peak aortic jet velocity (2.4 [2.0–2.7] versus 2.7 [2.4–3.0] m/s; *p* = 0.03) and larger effective orifice area (1.5 [1.3–1.8] versus 1.1 [1.0–1.5] cm^2^; *p* = 0.02) than patients with SAVR. But evidence of SVD appeared similar in TAVR and SAVR groups on echocardiography (6% versus 8%, respectively; *p* = 0.78) and CT (15% versus 14%, respectively; *p* = 0.87) [73]. The overall prevalence of patients with increased leaflet ^18^F-NaF uptake appeared nearly double in patients with SAVR (29% versus 15% in those with TAVR), but this was not statistically significant (*p* = 0.09). Therefore, across the three distinct imaging modalities, TAVR and SAVR bioprosthetic degeneration appear to be of similar magnitude, suggesting comparable durability. On univariable analysis, this study also revealed that the only predicators of the annualized change in peak velocity were valve age (*p* = 0.035), abnormal CT findings (*p* = 0.006), and ^18^F-NaF leaflet uptake (*p* < 0.001). However, on multivariable analysis incorporating age, sex, duration of valve implantation, baseline peak prosthetic valve velocity, and abnormal CT findings, ^18^F-NaF uptake was the only predicator of annualized change in peak velocity (*p* < 0.001). A relevant limitation of this study includes the small sample size. Patients with SAVR and TAVR were also not matched for age or comorbidities [73].

Four-year outcomes from the Evolut Low Risk Trial, a study that included patients who underwent either TAVR with a self-expanding supra-annular CoreValve/Evolut R/Pro (Medtronic) or SAVR, demonstrated no differences in valve reintervention (1.3% vs. 1.7%, *p* = 0.63), but an improved hemodynamic profile for TAVR patients including a decreased mean gradient (9.8 vs. 12.1 mm Hg, *p* < 0.001) and increased aortic valve area (2.1 vs. 2.0 cm^2^, *p* < 0.001).

Ten-year outcomes were studied in the Nordic Aortic Valve Intervention Trial (NOTION), a multicenter randomized clinical trial conducted across three sites in Scandinavia. The trial included 280 patients with severe aortic stenosis (145 treated with TAVR and 135 with SAVR), all aged 70 or older with low surgical risk, from 2009 to 2013. Patients in need of acute treatment or those with concomitant significant cardiovascular diseases and/or other major organ failures were excluded. Patients who underwent TAVR received a self-expanding CoreValve bioprosthesis, while those undergoing SAVR received a standard porcine or bovine stented bioprosthesis. Outcomes studied included bioprosthesis durability, classified according to the VARC-3 criteria, which included bioprosthetic valve failure (BVF) and bioprosthetic valve dysfunction (BVD). SVD was classified as a subset of BVD. Echocardiography outcomes were also studied, including the EOA of the bioprosthesis, the mean transprosthetic gradient, the degree of central regurgitation, and PVL. Results showed that TAVR patients had a higher rate of moderate or severe PVL or bioprosthesis regurgitation (TAVR 25.4% vs. SAVR 2.5%, *p* < 0.01). The risk of moderate or severe SVD was similar between the groups (TAVR 15.4% vs. SAVR 20.8%, HR 0.7; 95% CI 0.4–1.3, *p* = 0.3), while the risk of severe SVD was lower in TAVR patients (TAVR 1.5% vs. SAVR 10%, HR 0.2; 95% CI 0.04–0.7, *p* = 0.02). Severe BVD was less frequent in the TAVR group (TAVR 20.5% vs. SAVR 43.0%, *p* < 0.01), and overall, there was no significant difference in BVF between the groups (TAVR 9.7% vs. SAVR 13.8%, HR 0.7; 95% CI 0.4–1.5, *p* = 0.4) [74]. 

The current literature presents conflicting findings on the difference in risk for degeneration between TAVR and SAVR, suggesting that TAVR may be comparable to its surgical counterparts. Long-term risks of degeneration post-TAVR are yet to be established. Results from the 10-year follow up of the PARTNER 3 (a RCTS evaluating low-risk surgical patients) and Evolut Low Risk Trial will provide more conclusive evidence regarding SVD in TAVR vs. SAVR in the long-term setting [68].

## 7. Management of Bioprosthetic Aortic Valve Degeneration

Typical guidelines for surgical aortic valve replacement call for a mechanical aortic valve in younger patients (<50 USA, <65 Europe), bioprosthetic aortic valve in older patients (>70 USA, >65 Europe), or either option for ages in between. As of late, there has been greater consideration toward the use of biological valve replacement in younger patients due to the need of anticoagulation following mechanical valve replacement [11].

Structural valve degeneration remains the leading cause of aortic valve degeneration following replacement due to calcification and disruption of the leaflet. Glutaraldehyde is commonly used in graft preservation which crosslinks and masks xenograft antigens and makes it immunologically inert. Pre-treated valves with glutaraldehyde can still induce an immune response due to any residual Xeno antigens and can result in calcification. The current method that addresses SVD focuses on reducing calcification such as with the use of urazole or epoxy compounds. Anticalcification methods have enhanced valve durability beyond what is achieved with tissue fixation alone and typically involve using detergents to eliminate calcium-binding phospholipids or applying compounds that modify the structure of exposed aldehyde groups [75]. Alternative approaches suggest immune response modulation through genetic modification of xenografts and physical or detergent-based decellularization methods. These preventative methods show promise but have limitations in cost and risk to graft function. Long term anticoagulation past 3–6 months, immunosuppressives, and matrix metalloproteinase (MMP) following BAV have been shown to manifest in adverse side effects while statin therapy has shown promise but lacks long-term follow up [3].

**Figure 3:**
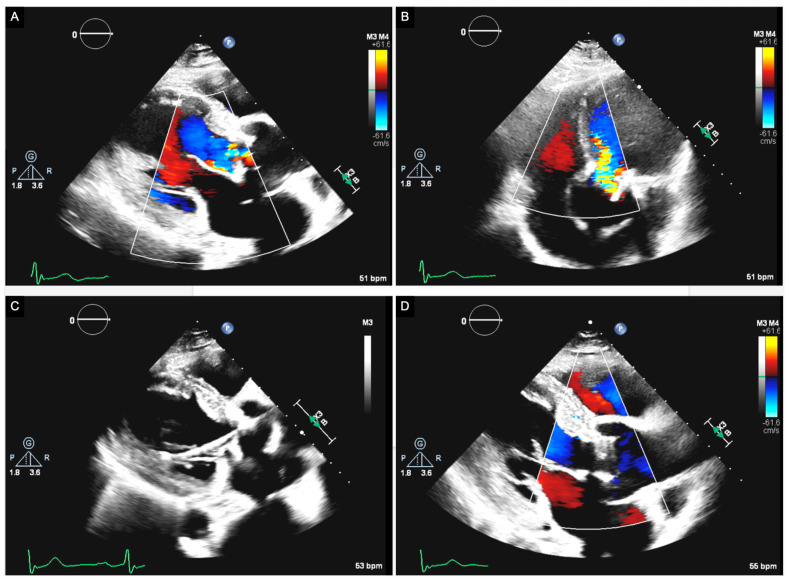
A 77-year-old male with a history of SAVR ten years ago. The patient developed severe SVD, and a valve-in-valve TAVR procedure was indicated. In the preoperative echocardiographic images (Panels **A**,**B**), moderate aortic valve stenosis and severe aortic valve regurgitation were observed. A 26 mm Edwards Sapien 3 Ultra transcatheter aortic valve bioprosthesis was implanted via a transfemoral approach (Panel **C**). Following the procedure (Panel **D**), a normal aortic prosthesis was observed with normal gradients (14 mmHg) and no prosthetic or periprosthetic aortic valve regurgitation.

Two current methods exist to address a failed BAV due to SVD: ViV TAVR or redo SAVR. As of 2017, redo SAVR was the most common approach to SVD, but ViV TAVR has begun to gain traction as an alternative for higher-risk patients [15] (Figure 3). BAV degeneration presents a challenging question when faced with the decision to undergo ViV TAVR or redo SAVR [76].

ViV TAVR involves placing a new transcatheter valve within an existing degenerated bioprosthesis. This method is less invasive with shorter operative time and greater short-term outcomes than the more standardly used redo SAVR. A 2009–2019 study involved 90 patients, with 73 undergoing ViV TAVR and 17 undergoing redo SAVR following degeneration of the BAV, with the aim of evaluating short term outcomes of both managements. ViV TAVR involved transfemoral (62 patients), transaortic (4 patients), or transapical (7 patients) implantation while redo SAVR was performed via median sternotomy [77]. The study found that the in-hospital all-cause mortality was significantly higher for redo SAVR than in the ViV TAVR group (17.6% vs. 0%, *p* < 0.001). The patients in the redo SAVR group also had a longer in-hospital stay than valve-in-valve TAVR patients (12.3 ± 7.3 vs. 10.6 ± 9.2 days, *p* = 0.316). Although the patients undergoing valve-in-valve TAVR had fewer postoperative complications, a greater chance of PVL was observed (52.1% compared to 0%, *p* < 0.001), with an overall higher transvalvular gradient on echocardiogram, aortic valve regurgitation, and vascular complications, when compared to redo SAVR patients [76,77]. Both groups showed similar rates of acute kidney injury (AKI) (34.7 vs. 29.4%, *p* = 0.677), with no significant difference in the rate of new dialysis (5.5 vs. 5.9%, *p* = 0.948) [77]. 

S. Yousef et el in a retrospective study compared the outcomes of 198 patients who underwent ViV TAVR (2013–2022) with 147 patients who underwent redo SAVR (2011–2022). The length of hospital stay was significantly higher in the SAVR group (8.0 (5.0–14.0) vs. 2.0 (1.0–6.0), *p* < 0.001) than in the ViV TAVR group. Operative mortality was low in both the groups (2%), but the observed-to-expected operative mortality (1.2 vs. 0.32) was higher in the SAVR group. Despite their substantially increased baseline risk, patients in the ViV TAVR group had fewer postprocedural complications [76].

A multicenter retrospective study in the UK included 911 patients who underwent redo SAVR and 411 patients who underwent ViV TAVR from July 2005 to April 2021 due to SVD [78]. In-hospital mortality was 7.2% (n = 9) for redo SAVR versus 0 for ViV TAVR, *p* = 0.002. Surgical patients suffered more postoperative complications, including intra-aortic balloon pump support (*p* = 0.02), early re-operation (*p* < 0.001), arrhythmias (*p* < 0.001), respiratory and neurological complications (*p* = 0.02 and *p* = 0.03), and multi-organ failure (*p* = 0.01). The ViV TAVR group had a shorter intensive care unit and hospital stay (*p* < 0.001 for both). However, moderate aortic regurgitation at discharge and higher postprocedural gradients were more common after ViV TAVR (*p* < 0.001 for both). Survival probabilities in patients who were successfully discharged from the hospital were similar after redo SAVR compared to ViV TAVR over the 6-year follow-up (92 ± 2.5% vs. 100% at 1 year, 90 ± 2.9% vs. 98 ± 1.3% at 2-year, 87 ± 3.4% vs. 94 ± 2.5% at 3-year, 80 ± 4.5% vs. 89 ± 3.9% at 4-year, 70 ± 5.5% vs. 77 ± 5.9% at 5-year, and 68 ± 5.8% vs. 67 ± 7.6% at 6-year follow-up, log-rank *p* = 0.26) [78]. Although the long-term results are limited and patient cohorts are small, several studies report the feasibility of ViV TAVR compared with redo SAVR for severe SVD [79,80,81].

In another study evaluating data from 2012 to 2016, with a large cohort including 1420 matched patients (710 ViV TAVR and 710 redo SAVR), similar results were found. The primary outcomes of interest were in-hospital adverse events composite outcome (mortality, myocardial infarction, stroke, or acute kidney injury). ViV TAVR was associated with lower in-hospital composite adverse outcomes (14.1% vs. 25.4%, *p* = 0.018) and a trend towards lower mortality (<1.0% vs. 5.2%; *p* = 0.06). ViV TAVR also showed a decreased length of hospital stay (mean 6.6 vs. 9.7 days; *p* < 0.01). Postoperative bleeding and transfusions were significantly lower for ViV TAVR (17.6% vs. 31.0% and 12% vs. 31%, respectively, *p* < 0.01 for both) [82].

A meta-analysis of six retrospective cohort studies including 498 patients concluded no statistically significant differences in early (30 days or in hospital) (OR, 0.91; *p* = 0.83) and midterm (180 days–3 years) all-cause mortality (OR, 1.42; = 0.21) between the ViV TAVR and redo SAVR groups. Due to the inclusion of retrospective studies, patients in the ViV TAVR group were significantly older and had a more frequent history of prior coronary artery bypass surgery [83].

Another meta-analysis from 2021 included 12 publications and 16,207 patients (ViV TAVR, n = 8048; redo SAVR, n = 8159). ViV TAVR showed lower rates of early (30-day) mortality (OR: 0.42; 95% CI: 0.28 to 0.63; *p* = 0.003), stroke (OR: 0.65; 95% CI: 0.55 to 0.76; *p* < 0.001), permanent pacemaker implantation (OR: 0.73; 95% CI: 0.22 to 2.43; *p* = 0.536), and major bleeding (OR: 0.49; 95% CI: 0.26 to 0.93; *p* = 0.034). Hospitalization was also shorter in the ViV TAVR group. On the other hand, ViV TAVR was associated with increased risk of myocardial infarction (OR: 1.50; 95% CI: 1.01 to 2.23; *p* = 0.045) and severe patient–prosthesis mismatch (OR: 4.63; 95% CI: 3.05 to 7.03; *p* < 0.001) [84].

A similar meta-analysis from 2021 including 15 studies and 8881 patients (4458 underwent ViV TAVR and 4423 redo SAVR) demonstrated no observable difference in stroke, MI, or pacemaker implantation between ViV TAVR and redo SAVR but did find a lower rate of AKI in ViV TAVR. Short-term mortality was lower in ViV TAVR (2.8% versus 5.0%, *p* = 0.02). Midterm mortality did not differ between groups, thus highlighting the potential benefit in short-term mortality for ViV TAVR compared with redo SAVR with no clear benefits regarding mid-term survival [85].

A recent meta-analysis of reconstructed time-to-event data from Kaplan–Meier curves was conducted by including nonrandomized studies published by 30 September 2021. This publication included ten studies with a total of 3345 patients (1676 ViV TAVR and 1669 redo SAVR). Along with prior publications, ViV TAVR confirmed a lower risk of early all-cause mortality in the first 44 days (HRH 0.67, 95% CI 0.49–0.93, *p* = 0.017). However, a reversal was observed in the HR after 197 days favoring redo SAVR (HR 1.53; 95% CI 1.22–1.93; *p* < 0.001). This trend was confirmed when analyzing only the matched populations (1143 pairs). Interestingly, this study underscores that the initial protective effect of ViV TAVR may reverse over time, with redo SAVR being protective in the mid–long-term follow-up [86].

The above-mentioned results only include data from observational studies; therefore, they should be interpreted with caution, and randomized trials are still warranted to define the best strategy for patients with SVD.

Personalized treatment is crucial to refine management techniques as it is important to note that ViV TAVR is best for high-surgical-risk patients and redo SAVR is best for patients with endocarditis, at risk for patient prosthesis mismatch, or with poor valve access. It is important to also realize that the patient’s anatomy sometimes dictates which options are available and can be a factor in the decision of which procedure a patient undergoes. For some patients, their anatomy is prohibitive for a ViV TAVR, and their only option is a redo SAVR. Usually this is due to coronary anatomy (heights above the aortic valve annulus) or a small effaced aortic root, again with risk of coronary obstruction.

The explantation of transcatheter valves is becoming increasingly common, both in planned and emergency situations. The primary reasons for these procedures are the deterioration or failure of the prosthesis and injuries caused during the initial implantation. Patients who need TAVR explantation face high mortality rates. Specifically, the mortality rate is approximately 12% for valve-in-valve TAVR explantations and between 14% and 17% for overall TAVR explantations. This is significantly higher compared to the 6% to 9% mortality rate for patients undergoing a second SAVR. These findings suggest that TAVR procedures, including valve-in-valve TAVRs, may not be as long-lasting as initially expected and could lead to higher mortality rates in subsequent interventions. Transcatheter valve therapies are often ideal for elderly and high-risk patients who cannot undergo surgery. However, for younger patients who are likely to outlive their transcatheter devices, the potential risks of future explantation surgeries must be carefully weighed. Therefore, multidisciplinary heart teams should thoroughly consider these risks when planning long-term management strategies for patients with cardiac valvular disease [87].

## 8. Conclusions

The field of bioprosthetic valve structural degeneration continues to evolve with a deeper understanding of pathophysiology, contributing factors, and therapeutical options for its management. Our review delineates the critical aspects of screening and diagnosing bioprosthetic valve degeneration, emphasizing the need for regular follow-up and advanced imaging techniques to ensure timely intervention. The comparative analysis of SAVR and TAVR reveals distinct advantages and limitations associated with each approach. While SAVR remains a robust option with a proven track record, TAVR offers a less invasive alternative with promising outcomes and similar rates of degeneration. Ultimately, the choice between SAVR and TAVR should be tailored to individual patient profiles, balancing factors such as valve durability, procedural risks, and patient preferences. Future research should continue to refine our understanding of bioprosthetic valve degeneration, enhance screening methods, and improve treatment strategies to further improve patient outcomes. As technology advances and more data becomes available, both SAVR and TAVR will continue to evolve, offering even better solutions for those in need of aortic valve replacement or reintervention.
